# Physical therapy students’ perception of their ability of clinical and clinical decision-making skills enhanced after simulation-based learning courses in the United States: a repeated measures design

**DOI:** 10.3352/jeehp.2022.19.34

**Published:** 2022-12-19

**Authors:** Fabian Bizama, Mansoor Alameri, Kristy Jean Demers, Derrick Ferguson Campbell

**Affiliations:** College of Rehabilitation Sciences, Doctor of Physical Therapy Program, University of St. Augustine for Health Sciences, Austin, TX, USA; Hallym University, Korea

**Keywords:** : Clinical decision-making, Clinical competence, Physical therapy, Self-efficacy, Simulation

## Abstract

**Purpose:**

It aimed to investigate physical therapy students’ perception of their ability of clinical and clinical decision-making skills after a simulation-based learning course in the United States.

**Methods:**

Survey questionnaires were administered to voluntary participants, including 44 second and third-year physical therapy students of the University of St. Augustine for Health Sciences during 2021–2022. Thirty-six questionnaire items consisted of 4 demographic items, 1 general evaluation, 21 test items for clinical decision-making skills, and 4 clinical skill items. Descriptive and inferential statistics evaluated differences in students’ perception of their ability in clinical decision-making and clinical skills, pre- and post-simulation, and post-first clinical experience during 2021–2022.

**Results:**

Friedman test revealed a significant increase from pre- to post-simulation in perception of the ability of clinical and clinical decision-making skills total tool score (P<0.001), clinical decision-making 21-item score (P<0.001), and clinical skills score (P<0.001). No significant differences were found between post-simulation and post-first clinical experience. Post-hoc tests indicated a significant difference between pre-simulation and post-simulation (P<0.001) and between pre-simulation and post-first clinical experience (P<0.001). Forty-three students (97.6%) either strongly agreed (59.1%) or agreed (38.5%) that simulation was a valuable learning experience.

**Conclusion:**

The above findings suggest that simulation-based learning helped students begin their first clinical experience with enhanced clinical and clinical decision-making skills.

## Introduction

### Background/rationale

The American Physical Therapy Association identifies clinical decision-making as vital to autonomous physical therapist practice [1]. Clinical reasoning and clinical decision-making skills are essential for completing entry-level physical therapy programs [2]. The importance of creating learning opportunities for clinical reasoning and execution of clinical decisions before students participate in clinical experiences is well-established [3,4]. To effectively prepare students for the first clinical experience, entry-level physical therapy programs have a call to action to promote best practices in developing clinical decision-making skills. Simulation-based learning (SBL) is a form of experiential learning that offers students real-world opportunities to develop and practice knowledge skills in an interactive simulated environment [5]. The rising use of SBL in entry-level physical therapy curriculum allows physical therapy students to develop clinical decision-making skills before the first clinical experience [6].

The impact of SBL on physical therapy students’ clinical decision-making skills across practice settings needs to be well-documented [7]. Previous literature supports SBL as an effective curriculum to promote physical therapy students’ knowledge translation to clinical practice in acute care settings [8]. However, there remains to be a gap in understanding the impact of SBL on the development of physical therapy students’ clinical decision-making skills across other practice settings [9].

### Objectives

The purpose of this study was to investigate the physical therapy students’ perception of their ability of clinical and clinical decision-making skills before and after a SBL course. The students’ perception was measured by their ability of clinical and clinical decision-making skills. We hypothesized that students’ perception of ability scores for clinical and clinical decision-making skills would increase after they progressed through SBL and their first clinical experience.

## Methods

### Ethics statement

This study protocol was approved by the University of St. Augustine for Health Sciences Institutional Review Board (IRB #PT-0623-300). Informed written consent was obtained from all participants.

### Study design

A single-group repeated measures design was employed using a survey questionnaire administered during pre-simulation, post-simulation, and post-first clinical experience.

### Setting

Participants were recruited from the University of St. Augustine for Health Sciences before the 8-week patient care management SBL course from September 2021 to August 2022. The course included faculty-led patient-actor case-based SBL with scenarios in outpatient and community-based settings with the application of clinical reasoning using the patient-client management model [3]. Students received a minimum of 30 hours of SBL opportunity with lab activities, including pre-briefing, simulation, debriefing, and summative final practical (Supplement 1A, B). A pre-course survey was sent on the first day of the SBL course, an after-course survey last day of the SBL course, and a post-first clinical experience survey last day after the clinical experience. Surveys were completed anonymously via SurveyMonkey, available from: https://www.surveymonkey.com. Respondents received no incentives for participation. Participants’ anonymity was maintained, with personal identifying information removed from all data before being handled by the primary investigator for data analysis.

### Participants

After written consent was obtained from 154 target participants, a web survey link to the questionnaire was sent to physical therapy students’ email addresses. Surveys were administered before an SBL course (pre-simulation), after an SBL course (post-simulation), and after the first clinical experience across 3 cohorts enrolled in an SBL course. The SBL course was offered in September 2021, January 2022, and May 2022. A pre-course survey was sent on the first day of the SBL course, an after-course survey was sent last day of the SBL course, and the post-first clinical experience survey was sent on the last day of the clinical experience. Respondents first read the questionnaire description and had the opportunity to provide informed consent to access. Targeted participants included only students enrolled in the SBL course and first clinical experiences in either the second year of a 3-year residential program or the third year of a 4-year flexible program curriculum. Physical therapy students not enrolled in SBL and their first clinical experiences were excluded from participating. A total of 44 (28.6%) of the 154 qualified target physical therapy students agreed to complete the questionnaire during pre-simulation, post-simulation, and post-first clinical experience.

### Variables

Variables were 21 items for participants’ perception of their ability of clinical decision-making skills and 4 items for clinical skills.

### Data sources/measurement

We developed an electronic survey questionnaire using concepts from published studies on the perception of the ability of physical therapy clinical decision-making and clinical skills (Supplement 2) [10].

The questionnaire consisted of 36 items and began with 4 demographic questions and 1 query about the benefit of SBL in the development of clinical decision-making skills. Next, the 21-item on students’ perception of their ability of clinical decision-making skills and 4 items on clinical skills. After that, there were 3 open questions and 3 items on the clinical experiences. Measurement items of the ability of clinical decision-making and clinical skills consisted of a 5-point Likert scale (“strongly disagree” to “strongly agree”).

Brudvig et al. [10] established the validity and reliability of the 25-item perception of the ability of clinical and clinical decision-making skills measurement scale. The scale demonstrated high reliability between the items, with a Cronbach α coefficient of 0.988 for the total test score, 0.984 clinical decision-making scale, and 0.964 for the clinical skills scale [10]. In the current study, based on the 95% confidence interval, test re-test reliability was supported with an intraclass correlation coefficient of 0.860. Cronbach α coefficient was 0.975 for the total test score, 0.971 for the clinical decision-making skills scale, and 0.922 for the clinical skills scale. The face and construct validity of the scale was supported as the median scores across clinical decision-making skills and clinical skills domains increased as students progressed through the curriculum, with narrowed variability of the score after the first clinical experience. After the first clinical experience, we anticipate less variance in student scores as student self-evaluation skills should improve [10,11]. Raw response data from 44 participants are available from Dataset 1.

### Bias

We acknowledge that survey questionnaire response bias could result in overestimating results. Three experts knowledgeable in survey methodology and publication records reviewed the questionnaire for question clarity, feasibility, scale-item reliability, and face validity to reduce response bias. Since participants are voluntary-based, there may be some selection bias.

### Study size

Post-hoc tests with Bonferroni correction for multiple tests raised confidence in our sample size to detect significant differences, and a power analysis for ANOVA repeated measures between factors indicated statistical power approximating 1.0 with an alpha of .05 and a large effect size (d = 0.80).

### Statistics

Data were analyzed using Excel ver. 2016 (Microsoft Corp., Redmond, WA, USA) and IBM SPSS ver. 28.0 (IBM Corp., Armonk, NY, USA). Descriptive statistics summarized the distribution, central tendency, and dispersion of responses. Friedman tests evaluated repeated measures of within-subject differences in physical therapy students’ perception of their ability of clinical and clinical decision-making skills at pre-SBL, post-simulation, and post-first clinical experience. Significance was set at α=0.05.

## Results

### Participants

**Fig. 1.**
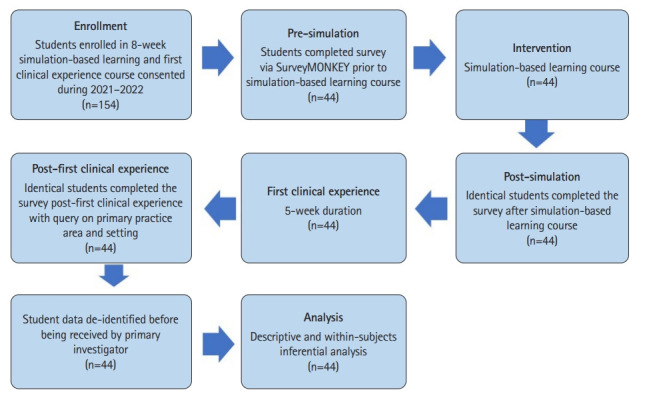
Flow chart of the survey on the physical therapy students’ perception of their ability of clinical and clinical decision-making skills in the United States.

A total of 44 physical therapy students (52.3% female) completed the questionnaire pre- and post-simulation and post-first clinical experience (response rate=28.6%). The largest proportion of respondents reported first clinical experience as primary area of clinical practice as orthopedics (71.4%) and primary clinical practice setting as an outpatient clinic (76.2%) (Fig. 1). Table 1 displays respondents’ demographic characteristics.

### Main results

#### Simulation-based learning as a valuable learning experience

Of the 44 physical therapy student respondents, 37 reported being in the second year of a 3-year traditional residential program, while 7 reported being in the third year of a 4-year alternative flexible entry-level physical therapy program. Overall, 97.6% of respondents either strongly agreed (59.1%) or agreed (38.5%) that simulation was a valuable educational learning experience to improve clinical decision-making skills, while 2.3% strongly disagreed.

#### Perception of the ability of clinical decision-making skills

**Fig. 2.**
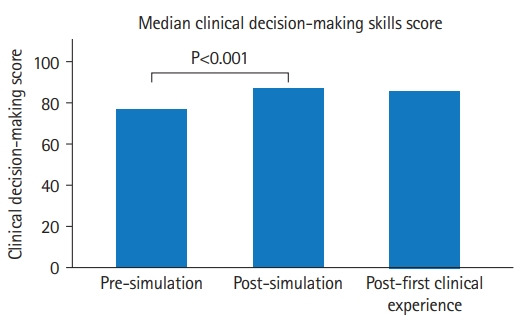
Score of measurement of physical therapy students’ perception of the ability of clinical decision-making skills at 3 consecutive times in the United States (N=44).

The Friedman test results indicated a difference in the clinical decision-making skills score across the 3-time points (P<0.001). Inspection of the median values showed an increase in score from pre-simulation (median=76.5; interquartile range [IQR], 67.5–84.0) to post-simulation (median=87.0; IQR, 79.5–99.5) and from pre-simulation (median=76.5; IQR, 67.5–84.0) to post-first clinical experience (median=85.5; IQR, 83.0–99.3). Post-hoc tests with Bonferroni correction indicated that there was a significant difference in scores between pre-simulation and post-simulation (P<0.001) and between pre-simulation and post-first clinical experience (P<0.001). There was no significant difference between post-simulation to post-first clinical experience scores (Fig. 2, Table 2).

#### Perception of the ability of clinical skills

**Fig. 3.**
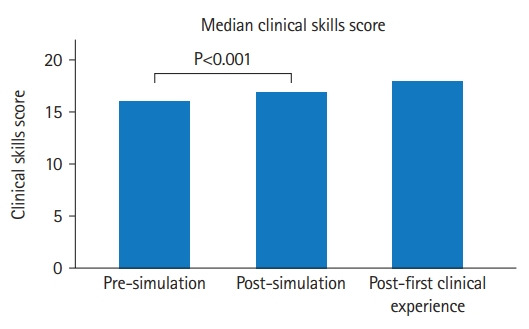
Score of measurement of physical therapy students’ perception of the ability of clinical skills at 3 consecutive times in the United States (N=44).

The Friedman test results indicated a difference in the clinical skills 4-item scale score across the 3-time points at pre-simulation, post-simulation, and post-first clinical experience (P<0.001). Inspection of the median values showed an increase in clinical skills score from pre-simulation (median=16; IQR, 14–16) to post-simulation (median=17; IQR, 16–20), and from pre-simulation (median=16; IQR, 14–16) to post-first clinical experience (median=18; IQR, 16–20). Post-hoc tests with Bonferroni correction indicated that there was a significant difference in clinical skills score between pre-simulation and post-simulation (P<0.001) and between pre-simulation and post-first clinical experience (P<0.001). There was no significant difference between post-simulation to post-first clinical experience clinical skills scale scores (Fig. 3, Table 2).

## Discussion

### Key results

The aim was to investigate if students’ perception of ability scores for clinical and clinical decision-making skills increase after a SBL course. Physical therapy students’ scores of perception of ability of clinical and clinical decision-making skills increased after progression through SBL and the first clinical experience. Furthermore, most physical therapy students believed SBL to be a valuable educational learning intervention to improve clinical decision-making skills.

### Interpretations

Our study found a significant increase with a large effect size from pre- to post-simulation in score of perception of ability of clinical and clinical decision-making but not between post-simulation and post-first clinical experience. These findings suggest that a ceiling effect on the development of physical therapy students’ clinical decision-making skills may occur since no significant difference was found between post-simulation and post-first clinical experience.

### Comparison with previous studies

Findings are similar to previous literature supporting the use of SBL to enhance the development of physical therapy students’ clinical decision-making skills in acute care practice settings [9]. Our findings suggest that SBL may enhance student clinical decision-making skills across outpatient and community-based practice settings. Our response rate (28.6%) was higher than the minimal recommended college student questionnaire response rate range (20%–25%) to allow for increased confidence in estimates [12].

### Limitations and generalizability

We suggest a randomized controlled study to strengthen support inference about causation. The study used a convenience sample and therefore is limited in its generalizability. We recommend replicating the study with a larger sample size across public and private institutions, and across increased practice-setting representation to confirm the external validity of our results. Since we invited 3 consecutive university student cohort populations pre- and post-SBL and post-first clinical experience to participate in the questionnaire, rather than a one-time sample, our response rate was sufficient to draw reasonable conclusions. These results may apply to physical therapy students in other institutes in the United States.

### Suggestions

Future research should investigate whether the ceiling effect on physical therapy students’ clinical decision-making development remains between first and final clinical experiences. Since questionnaire items assessed in our investigation are not unique to physical therapy, it may be beneficial to evaluate the SBL programs of other health professions using this scale.

### Implications for practice

Our findings suggest that SBL may be helpful in the development of physical therapy students’ clinical decision-making skills across outpatient and community-based practice settings [13]. Students should demonstrate clinical reasoning competencies before entering clinical experiences [4,5]. Use of the perception of the ability of clinical and clinical-decision-making skills tool may identify students with lower clinical decision-making and provide remediation opportunities before and during clinical experiences. With the future footprint of SBL in entry-level physical therapy programs expected to increase, developing students’ clinical decision-making skills is critical for the best practice. Our study emphasizes the value of physical therapy students’ perceptions of the ability of clinical decision-making and clinical skills before and after clinical experiences.

### Conclusion

Investigating physical therapy students’ perception of their ability of clinical and clinical decision-making skills may guide academic curricula in best practices to facilitate clinical readiness. Significant score increases in students’ perception of their ability of clinical and clinical decision-making skills were found between pre-simulation and post-simulation in curriculum, but not between post-simulation and post-first clinical experience. These findings suggest that SBL was helpful for students to prepare for their first clinical experience with enhanced clinical and clinical decision-making skills. We recommend considering expanding SBL in entry-level physical therapy programs to enhance clinical decision-making skills before clinical experiences.

## Figures and Tables

**Table 1.** Demographic data of respondents (n=44)

Characteristic	Value
Age (yr)	26±4.0
Gender	
Female	23 (52.3)
Male	20 (46.5)
Prefer not to answer	1 (2.3)
Program	
Residential	37 (84.1)
Flex	7 (15.9)
Race/ethnicity	
American or Alaskan Indian	3 (6.8)
Asian/Pacific Islander	6 (13.6)
Black or African American	4 (9.1)
Hispanic	6 (13.6)
White Caucasian	22 (50.0)
Prefer not to answer	0
Multiple ethnicity/other	3 (0)
Area of clinical practice	
Orthopedics	30 (71.4)
Neurorehabilitation	4 (9.5)
Other	8 (19.0)
Practice	
Outpatient clinic	32 (76.2)
Home health	1 (2.4)
Skilled nursing facility	3 (7.1)
Inpatient hospital	2 (4.8)
Inpatient rehabilitation facility	4 (9.5)
Other	0

Values are presented as mean±standard deviation or number (%).

**Table 2.** Friedman pairwise comparison test results for single group repeated measures (n=44)

Within subjects tested	CDM score			CS score		
	χ^2^	df	P-value	χ^2^	df	P-value
Pre-Sim vs. Post-Sim	37.377	2	<0.001	33.922	2	<0.001
Post-Sim vs. Post-first clinical experience		2	0.456		2	0.915
Pre-Sim vs. Post-first clinical experience		2	<0.001		2	<0.001

CDM, clinical decision-making 21-item sub-scale; CS, clinical skills 4-item sub-scale; df, degrees of freedom; Pre-Sim, pre-simulation; Post-Sim, post-simulation.

## Footnotes

**Data availability**
Data files are available from Harvard Dataverse: https://doi.org/10.7910/DVN/WCNLIK Dataset 1. Raw response data from 44 participants at pre-simulation, post-simulation-based learning, and before clinical experience.

## Supplementary materials

Supplementary files are available from Harvard Dataverse: https://doi.org/10.7910/DVN/WCNLIK

Supplement 1. (A, B) Patient care management simulation-based learning course content.

Supplement 2. Measurement tools for physical therapy students’ perception of clinical and clinical decision-making skills.

Supplement 3. Audio recording of the abstract.
