# Supplementary material for: Physical therapy students’ perception of their ability of clinical and clinical decision-making skills enhanced after simulation-based learning courses in the United States: a repeated measures design
Source: J Educ Eval Health Prof. 2022 Dec 19;19:34. doi: 10.3352/jeehp.2022.19.34 (PMC9845066; doi:10.3352/jeehp.2022.19.34)
Supplement: Supplementary file 2 — Supplement 1. (A, B) Patient care management simulation-based learning course content. [file jeehp-19-34-suppl1.pdf]

Simulation-based learning content

- 10 Case-Based Lab Sessions
- Designed for 30-72 Physical Therapy (PT)Students
- Simulation Teams
  - ~10 Students/Team
  - Faculty Instructor
  - Student PT
  - PT Technician (Tech) or Physical Therapy Assistant (PTA)
  - Student Observers
- 2 Hour Lab Sessions:
  - 45-60 minutes summative simulation:
    - Physical Therapy evaluation or re-evaluation & treatment of a complex patient case in simulated outpatient setting
    - Plan of care development
    - Documentation
  - 45-60 minutes debriefing and Q&A
    - Student PT's self-reflection
    - Observer student feedback
    - Instructor feedback

*PCM III SIMULATION ROLE ASSIGNMENTS

Student Name	Simulation Team	Case 1.1 Lab 1	Case 2.1 Lab 2	Case 3.1 Lab 3	Case 4.1 Lab 4	Case 5.1 Lab 5	Case 1.2 Lab 6	Case 2.2 Lab 7	Case 3.2 Lab 8	Case 4.2 Lab 9	Case 5.2 Lab 10
Student #1	Blue	PT					PTA				
Student #2	Blue	PT Tech					PT				
Student #3	Blue		PT					PTA			
Student #4	Blue		PT Tech					PT			
Student #5	Blue			PT					PTA		
Student #6	Blue			PT Tech					PT		
Student #7	Blue					PT				PTA	
Student #8	Blue				PT Tech					PT	
Student #9	Blue				PT						PT Tech
Student #10	Blue					PT Tech					PT

Student Name	Simulation Team	Case 1.1 Lab 1	Case 2.1 Lab 2	Case 3.1 Lab 3	Case 4.1 Lab 4	Case 5.1 Lab 5	Case 1.2 Lab 6	Case 2.2 Lab 7	Case 3.2 Lab 8	Case 4.2 Lab 9	Case 5.2 Lab 10
Student #11	Tan	PT					PTA				
Student #12	Tan	PT Tech						PT			
Student #13	Tan		PT					PTA			
Student #14	Tan		PT Tech				PT				
Student #15	Tan			PT		PT Tech					
Student #16	Tan			PT Tech						PT	
Student #17	Tan				PT					PTA	
Student #18	Tan				PT Tech				PT		
Student #19	Tan					PT			PTA		

Student #	Simulation Team	Case 1.1 Lab 1	Case 2.1 Lab 2	Case 3.1 Lab 3	Case 4.1 Lab 4	Case 5.1 Lab 5	Case 1.2 Lab 6	Case 2.2 Lab 7	Case 3.2 Lab 8	Case 4.2 Lab 9	Case 5.2 Lab 10
Student #20	Green	PT					PTA				
Student #21	Green	PT Tech					PT				
Student #22	Green		PT					PTA			
Student #23	Green		PT Tech					PT			
Student #24	Green			PT					PTA		
Student #25	Green			PT Tech					PT		
Student #26	Green					PT				PTA	
Student #27	Green				PT Tech					PT	
Student #28	Green				PT						PT Tech
Student #29	Green					PT Tech					PT

*PCM=patient care management

Simulation-based learning course content: Case Scenarios

Initial Evaluations	Re-evaluations
**Case 1**: **Chronic stroke and end stage renal disease–**67-year-old Hispanic male receiving dialysis. Right spastic hemiplegia, neuropathy, HTN, and multiple medications. Received inpatient acute care for 1 week, 30-day skilled nursing facility rehabilitation, and 30-day home health rehabilitation.	**Case 6**: **Progression of right sided heart failure-**Patient complains of feet swelling over the past week. Physician ordered no salt diet and increased dosage of diuretic.
**Case 2**: **COPD and heart failure-**58-year old Caucasian male with a chronic history of COPD, hypertension, dyslipidemia, coronary artery disease. Attends cardiac rehabilitation on a 1-time weekly basis.	**Case 7**: **Progression of left sided heart failure secondary to medication non-compliance**-The patient is very argumentative about trying to stop smoking.
**Case 3**: **Type 2 diabetes & chronic pain-**63-year old African-American male, BMI of 33, with small ulceration between Right great and ring toe webbing, stage II wound; no dressing. Has had flashbacks from war. Walks slower than normal, complains that feet hurt regardless of walking or sitting.	**Case 8**: **Trans-metatarsal amputation, causalgia, & phantom limb pain**-The patient developed gangrene in all toes underwent a trans-metatarsal amputation 4 weeks ago. He is full weight-bearing using a single-point cane and shoe with filler.
**Case 4: Lymphedema, cancer rehab & chronic pain**-55-year old morbidly obese Caucasian female admitted to outpatient with severe loss of functional mobility and increased swelling to her lower extremities. History of Left breast cancer, axillary desmoid tumor, and chronic pain.	**Case 9**: **Acute cellulitis and lymphedema-**Left upper extremity and chest wall. The surgeon had to remove the breast implant. Progression of upper body chronic pain.
**Case 5**: **Osteoporosis, compression fracture & incontinence**-a 72-year old female, competitive walker diagnosed with compression fracture and urinary incontinence. Using a cruciform anterior spinal extension brace.	**Case 10**: **Post-hospitalization, UTI, dehydration and fear of falling**-Incontinence worse since hospitalization.
