# Supplementary material for: Physical therapy students’ perception of their ability of clinical and clinical decision-making skills enhanced after simulation-based learning courses in the United States: a repeated measures design
Source: J Educ Eval Health Prof. 2022 Dec 19;19:34. doi: 10.3352/jeehp.2022.19.34 (PMC9845066; doi:10.3352/jeehp.2022.19.34)
Supplement: Supplementary file 3 — Supplement 2. Measurement tools for physical therapy students’ perception of clinical and clinical decision-making skills. [file jeehp-19-34-suppl2.pdf]

* 1. What is your age?

* 2. What is your gender?

- Female
- Male
- Prefer not to answer
- Other (please specify)

* 3. Which race or ethnicity best describes you? (Please choose only one)

- American Indian or Alaska Native
- Asian/Pacific Islander
- Black or African American
- Hispanic or Latino
- White or Caucasian
- Prefer not to answer
- Multiple ethnicity/other (please specify)

* 4. Which Doctor of physical therapy (DPT) program at the University of St. Augustine for Health Sciences are you enrolled in?

- Residential DPT program
- Flex DPT program

* 5. Simulation was a valuable educational learning experience to improve my clinical decision-making skills.

- Strongly disagree
- Disagree
- Neither agree nor disagree
- Agree
- Strongly agree
- n/a

* 6. **The following questions aim to understand how each participant perceives their own abilities. Please rate the level of agreement on your ability to:**

Recognize physiological and psychological changes in patients in a timely manner and adjusts patient interventions
accordingly.

- Strongly disagree
- Disagree
- Neither agree nor disagree
- Agree
- Strongly agree

* 7. Synthesize information from multiple data sources to make clinical decisions (patient and caregiver, healthcare
professional, evidence and medical records).

○ Strongly disagree ○ Disagree ○ Neither agree nor disagree ○ Agree ○ Strongly agree

* 8. If information is not readily available, seek out the necessary information.

○ Strongly disagree ○ Disagree ○ Neither agree nor disagree ○ Agree ○ Strongly agree

* 9. Present a logical rationale (cogent and concise argument) for clinical decision.

○ Strongly disagree ○ Disagree ○ Neither agree nor disagree ○ Agree ○ Strongly agree

* 10. Demonstrate an ability to make clinical decisions in ambiguous situations.

○ Strongly disagree ○ Disagree ○ Neither agree nor disagree ○ Agree ○ Strongly agree

* 11. Select interventions based on best available evidence, clinical expertise, and patient preferences.

○ Strongly disagree ○ Disagree ○ Neither agree nor disagree ○ Agree ○ Strongly agree

* 12. Clinical decisions focus on the whole person rather than the disease.

○ Strongly disagree ○ Disagree ○ Neither agree nor disagree ○ Agree ○ Strongly agree

* 13. Identify clinical situations where physical therapy treatment is either not required or is contraindicated and
makes appropriate referral.

○ Strongly disagree ○ Disagree ○ Neither agree nor disagree ○ Agree ○ Strongly agree

* 14. Select tests and measures accurately and proficiently.

○ Strongly disagree ○ Disagree ○ Neither agree nor disagree ○ Agree ○ Strongly agree

* 15. Interpret tests and measurements accurately.

○ Strongly disagree ○ Disagree ○ Neither agree nor disagree ○ Agree ○ Strongly agree

* 16. Sequence tests and measures in logical manner to optimize efficiency.

○ Strongly disagree ○ Disagree ○ Neither agree nor disagree ○ Agree ○ Strongly agree

* 17. Adjust tests and measures according to the patient’s response.

○ Strongly disagree ○ Disagree ○ Neither agree nor disagree ○ Agree ○ Strongly agree

* 18. Synthesize examination data and identifies pertinent impairments, functional limitations, and quality of life.

○ Strongly disagree ○ Disagree ○ Neither agree nor disagree ○ Agree ○ Strongly agree

* 19. Reach clinical decisions efficiently

◯ Strongly disagree ◯ Disagree ◯ Neither agree nor disagree ◯ Agree ◯ Strongly agree

* 20. Integrate data and arrives at an accurate prognosis.

◯ Strongly disagree ◯ Disagree ◯ Neither agree nor disagree ◯ Agree ◯ Strongly agree

* 21. Estimate the contribution of factors (e.g., preexisting health status, co-morbidities, race, ethnicity, gender, age,
health behaviors) on the patient’s prognosis.

◯ Strongly disagree ◯ Disagree ◯ Neither agree nor disagree ◯ Agree ◯ Strongly agree

* 22. Establish a plan of care consistent with the examination and evaluation.

◯ Strongly disagree ◯ Disagree ◯ Neither agree nor disagree ◯ Agree ◯ Strongly agree

* 23. Re-evaluate and adjust the plan of care appropriately.

◯ Strongly disagree ◯ Disagree ◯ Neither agree nor disagree ◯ Agree ◯ Strongly agree

* 24. Provide rationale for interventions selected for patients with various diagnoses.

◯ Strongly disagree ◯ Disagree ◯ Neither agree nor disagree ◯ Agree ◯ Strongly agree

* 25. Use appropriate standardized outcome measures throughout a patient's episode of care.

◯ Strongly disagree ◯ Disagree ◯ Neither agree nor disagree ◯ Agree ◯ Strongly agree

* 26. Evaluate whether functional goals from the plan of care have been met.

◯ Strongly disagree ◯ Disagree ◯ Neither agree nor disagree ◯ Agree ◯ Strongly agree

* 27. Conduct tests and measures appropriately.

◯ Strongly disagree ◯ Disagree ◯ Neither agree nor disagree ◯ Agree ◯ Strongly agree

* 28. Perform interventions effectively.

◯ Strongly disagree ◯ Disagree ◯ Neither agree nor disagree ◯ Agree ◯ Strongly agree

* 29. Perform interventions efficiently.

◯ Strongly disagree ◯ Disagree ◯ Neither agree nor disagree ◯ Agree ◯ Strongly agree

* 30. Perform interventions in a coordinated manner.

◯ Strongly disagree ◯ Disagree ◯ Neither agree nor disagree ◯ Agree ◯ Strongly agree

* 31. What has been the most helpful element to develop your clinical decision making skills?

* 32. What has been the least helpful element to develop your clinical decision making skills?

* 33. Please describe your clinical skills and clinical decision-making in a few words.

34. Have you completed your Integrated Clinical Experience (ICE)?

- Yes
- No

* 35. What was the primary area of practice during your clinical experience?

- ○ Orthopedics
- ○ Neurorehabilitation
- ○ Other (please specify)

[ ]

* 36. What was the primary practice setting during your clinical experience?

- ○ Outpatient clinic
- ○ Home Health
- ○ Skilled Nursing Facility
- ○ Inpatient-Hospital
- ○ Inpatient Rehabilitation Facility
- ○ Other (please specify)

[ ]
